# Synthetic Cadaver Odorants and the Sulfur Gap: Linking Chemistry and Canine Olfaction in Human Remains Detection

**DOI:** 10.3390/molecules30204066

**Published:** 2025-10-13

**Authors:** Iwona Kowalczyk-Jabłońska, Bartłomiej Zieniuk, Magdalena Pawełkowicz

**Affiliations:** 1 Department of Animal Genetics and Conservation, Institute of Animal Sciences, Warsaw University of Life Sciences-SGGW, 8 Ciszewskiego Str., 02-786 Warsaw, Poland; iwona_kowalczyk-jablonska@sggw.edu.pl; 2 Department of Chemistry, Institute of Food Sciences, Warsaw University of Life Sciences-SGGW, 159C Nowoursynowska Str., 02-776 Warsaw, Poland; bartlomiej_zieniuk@sggw.edu.pl; 3 Department of Plant Genetics, Breeding and Biotechnology, Institute of Biology, Warsaw University of Life Sciences-SGGW, 159 Nowoursynowska Str., 02-776 Warsaw, Poland

**Keywords:** human remains detection dogs, search-and-rescue dogs, thanatochemistry, forensic science, synthetic cadaver odorants, canine olfaction, olfactory receptors (ORs), decomposition odor, sulfur gap hypothesis, odor signal transduction

## Abstract

Human remains detection (HRD) dogs are vital tools in forensic science and disaster response, but their training is limited by the restricted availability of human material. Synthetic odorants such as Sigma Pseudo™ formulations provide safer, standardized alternatives, yet current products reproduce only a fraction of the volatile organic compound (VOC) profile of decomposition. In particular, sulfur-containing volatiles, which are highly odor-active and consistently present in human remains, are often missing, reducing biological fidelity. Here, we integrate analytical chemistry with canine olfactory genetics and molecular biology to explain these limitations. Dogs possess one of the largest olfactory receptor (OR) repertoires among mammals, with high allelic diversity and specialized trace amine-associated receptors (TAARs) tuned to cadaveric amines. Together with olfactory binding proteins (OBPs) and ciliary signal transduction cascades, these molecular mechanisms highlight why incomplete VOC mixtures may fail to activate the full receptor network required for reliable odor imprinting. We propose the “sulfur gap hypothesis” and suggest hybrid training strategies combining improved synthetics with ethically sourced biological samples to enhance HRD dog performance.

## 1. Introduction

Human remains detection (HRD) dogs are invaluable assets in forensic science, frequently deployed to locate clandestine graves, recover remains in mass fatality incidents, and support criminal investigations where only trace amounts of decomposition odor are present [1,2]. Their extraordinary olfactory acuity enables them to detect volatile organic compounds (VOCs) associated with human decomposition at extremely low concentrations, making them uniquely effective in both forensic and search-and-rescue (SAR) contexts [3,4]. Depending on their operational specialization, dogs may be trained as SAR dogs for disaster scenarios or as HRD dogs dedicated to forensic contexts, where they must distinguish between live human scent and specific VOCs released during decomposition [5,6,7].

A substantial body of knowledge on decomposition processes originates from research conducted at specialized facilities such as the Anthropological Research Facility in Tennessee, which has enabled the identification of decomposition-specific VOCs across stages and environments [8,9]. Nevertheless, the use of human remains in training and research is restricted by strict ethical, legal, and biosafety regulations, both in the United States and internationally [10,11,12,13]. These constraints limit access to authentic biological material, creating variability in training opportunities and dog performance. As a result, there is growing interest in synthetic odorants, such as Sigma Pseudo™ Corpse Scent formulations, designed to replicate the odor profile of human decomposition while offering safe and standardized alternatives [14]. However, the accuracy of these synthetic substitutes and their ability to elicit reliable detection responses remain under scientific scrutiny [15].

Recent studies and reviews have emphasized the potential of synthetic odorants but also revealed significant challenges. Synthetic mixtures reproduce only part of the decomposition VOC spectrum, and behavioral studies show that dogs trained exclusively on synthetics may fail to generalize detection to authentic human remains [16,17]. A key limitation observed in the few synthetic training aids that have been chemically characterized is the apparent underrepresentation of sulfur-containing VOCs, such as dimethyl disulfide (DMDS) and dimethyl trisulfide (DMTS), which are consistently detected in decomposition headspace and are among the most odor-active compounds relevant to canine detection [8]. These findings raise questions about the biological fidelity of currently characterized formulations, although the extent to which this pattern applies across all commercial aids remains uncertain. Parallel advances in analytical chemistry, particularly GC–MS and GC × GC–TOFMS, have refined VOC characterization [18], while molecular studies reveal that dogs express over 1000 functional olfactory receptor (OR) genes, many tuned to sulfur- and nitrogen-based ligands [19,20]. These findings highlight the need to move beyond simple chemical mimicry toward biologically informed synthetic design.

Recent advances in canine genomics and molecular olfactory biology further expand the understanding of HRD dog performance beyond anatomical and behavioral frameworks. Dogs possess one of the largest olfactory receptor (OR) repertoires among mammals, with over 1000 functional genes organized in genomic clusters, providing the molecular foundation for their extraordinary scent detection capabilities [21,22]. High genetic variability and breed-specific allelic differences contribute to inter-individual variation in odor sensitivity [23], while expression studies reveal dynamic regulation of OR transcripts in the nasal epithelium [20]. In addition, trace amine-associated receptors (TAARs) complement classical ORs by detecting cadaveric amines such as putrescine and cadaverine, molecules strongly linked to decomposition odor [24,25]. Together with membrane-level processes involving olfactory binding proteins (OBPs) and ciliary signal transduction cascades [26,27], these genetic and molecular mechanisms provide a biologically grounded framework for evaluating synthetic odorants and identifying critical gaps—such as the absence of sulfur-containing volatiles—that limit their fidelity for HRD training.

**Figure 1:**
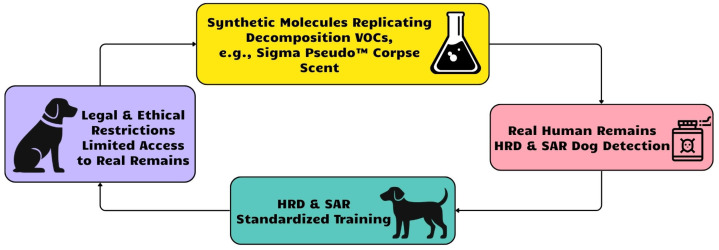
Synthetic molecules like Sigma replicate decomposition odors, enabling standardized training of forensic and rescue dogs.

The present study addresses these gaps by linking decomposition chemistry with canine olfactory biology. Specifically, we (i) critically evaluate the chemical fidelity of synthetic aids, with emphasis on missing sulfur volatiles; (ii) propose the sulfur gap hypothesis as a mechanistic explanation for limited behavioral transfer; (iii) introduce a receptor-guided framework for the rational design of synthetic training aids; and (iv) outline a hybrid training strategy that integrates improved synthetic mixtures with ethically sourced biological materials. Together, these contributions provide a biologically grounded pathway toward more standardized, effective, and ethical training practices for HRD and SAR dogs (Figure 1).

## 2. Chemical Composition of Decomposition Odor

### 2.1. Introduction to Thanatochemistry

The term “death” refers to the cessation of all vital biological functions in an organism. Following death, the process of decomposition begins, and the human body undergoes a transformation [28]. Scientific evidence confirms that irreversible cardiac arrest and halted cellular metabolism signal the beginning of decomposition [14]. The characteristic and often intense odor associated with decomposing organic material, particularly human remains, is a complex chemical signature produced by thanatochemical processes. Thanatochemistry (from Greek *Thanatos* [death] and chemistry) is the field studying chemical changes postmortem, crucially applied to determine the postmortem interval (PMI) [29]. This field is fundamental to understanding the origin and evolution of decomposition odors—critical in forensic taphonomy, search and rescue (e.g., cadaver dog training), and environmental science. The volatile organic compounds (VOCs) comprising this odor profile are systematic products of tissue breakdown by intrinsic enzymes (autolysis) and extrinsic microorganisms (predominantly bacteria and fungi) [30].

Key VOC classes include nitrogen- and sulfur-containing compounds, carboxylic acids, aldehydes, ketones, and alcohols. VOC profiles vary significantly due to factors including temperature, humidity, soil type, pH, oxygen availability, cause of death, body mass, insect activity, and scavenging [14,31]. For this reason, sampling and analytical techniques (e.g., Gas Chromatography-Mass Spectrometry, GC-MS) must be rigorously optimized to capture VOCs at trace concentrations in complex matrices. In non-targeted analysis, conventional 1D gas chromatography (GC) often fails to resolve all components; thus, multidimensional methods like comprehensive two-dimensional gas chromatography (GC × GC) enable more detailed characterization [28]. In parallel with these instrumental considerations, the cross-study synthesis in Table S1 highlights that matrix and method significantly influence the observed profiles: studies employing thermal desorption or GC × GC modalities typically report a broader, chemically diverse VOC ensemble than 1D GC-MS, regardless of biological variation [4,9,32,33,34,35,36,37,38,39,40,41,42,43,44,45,46,47,48,49,50,51,52,53,54,55] (Table S1).

### 2.2. Key Volatile Organic Compounds (VOCs) in Decomposition

Given the need for experimental models in decomposition research, the pig (*Sus scrofa*) is widely regarded as a suitable human analog due to its similar internal anatomy, fat distribution, chest cavity size, lack of heavy fur, and omnivorous diet, which may result in a comparable gut fauna. However, Raymer et al. [56] highlighted that human remains tend to produce more esters than animal remains, a key difference in VOCs during decomposition [56].

In a controlled field study conducted in an open eucalypt woodland on the Cumberland Plain in Western Sydney, Australia, Nizio et al. [57] buried six pig carcasses clothed in cotton T-shirts and exhumed them at 1, 3, 6, 12, 18, and 24 months post-burial to investigate decomposition processes and textile degradation under temperate environmental conditions. Textiles associated with the remains showed significant staining and tissue adhesion early on. Chemical analysis revealed a complex VOC profile in the experimental textiles, with hundreds of additional compounds detected compared to controls. Carboxylic acids dominated the VOC profile, while soft tissue was present but decreased dramatically as skeletonization occurred. Other classes such as esters, nitrogen-containing compounds, and aromatics also varied by decomposition stage. Sulfur-containing compounds, often linked to decomposition odor, were surprisingly scarce in the textiles, possibly due to their retention in the surrounding soil. The VOC profile in the experimental textiles diminished after 24 months, consistent with full skeletonization and advanced textile degradation [57].

The process of decomposition is often described in five stages: fresh, bloated, decay (active), post-decay (advanced), and dry [14,56].

The fresh stage (days 1–3) involves poisoning the cells with carbon dioxide and accumulated metabolites, leading to autolysis. Early postmortem changes such as *pallor mortis* (paleness), *livor mortis* (skin discoloration), *algor mortis* (body cooling), and *rigor mortis* (ATP depletion and lactic acid buildup in muscle cells, causing a rigid connection between myosin and actin) are observed [58]. In a study in Bécancour, Québec, Canada—a mixed temperate forest environment, Patel et al. [59] collected VOCs from three human cadavers placed on the soil surface under anti-scavenging cages. Sampling occurred daily over six-day trials in summer conditions (June–August 2021). VOCs detected during the fresh stage were predominantly ante-mortem, including compounds such as 6-methyl-5-hepten-2-one associated with skin microbiota and environmental exposure, along with nitrogen-containing, ester, and halogenated compounds. In addition, Thurn et al. [60], in a separate study at the Australian Facility for Taphonomic Experimental Research in western Sydney, used two human donors placed in open-air conditions within a dry sclerophyll forest. Their findings indicated that the fresh stage was dominated by ketones, terpenes, alcohols, and trace sulfur compounds like dimethyl sulfide (DMS), dimethyl disulfide (DMDS), and dimethyl trisulfide (DMTS).

The bloated stage (days 3–10) is characterized by anaerobic bacterial putrefaction, marked by the production of gases, bloating, discoloration, and a noticeable odor [14]. The breakdown of soft tissues releases foul-smelling gases, i.e., post-mortem VOCs, especially Patel et al. [59] observed significant increases in sulfur-containing VOCs (DMDS, DMTS) and nitrogen-containing compounds (e.g., benzonitrile, methenamine). Other studies also mentioned the increased concentrations of hydrocarbons, alcohols, esters, and sulfur compounds (notably carbon disulfide and DMDS) [14].

Active decay (days 7–20) involves extensive tissue breakdown during peak maggot activity and microbial growth, releasing fluids, increasing temperature, and emitting VOCs such as cadaverine and putrescine, resulting in “black putrefaction” [14]. The study of Thurn et al. [60], which employed thermal desorption and GC × GC analyses, also revealed that active decomposition under warm, temperate field conditions was associated with a significant increase in alcohols (especially phenol), esters, ketones, and sulfur compounds (DMDS, DMTS).

Advanced decay (days ~29–51) involves maggot pupation and the development of a nutrient-rich Cadaver Decomposition Island (CDI) in the soil. This period showed the highest number (725) and abundance of VOCs, including short-chain alcohols (e.g., 1-propanol, 1-butanol), ketones (2-butanone, 2-pentanone), and aldehydes (hexanal, octanal, nonanal), mostly originating from fat and tissue breakdown [60]. These results, however, reflect specific environmental conditions and a limited sample size and may not be generalizable to all climates. The origins of many postmortem VOCs remain unclear, however, most are produced through enzymatic and microbial processes during the decomposition of bodies. Volatile sulfur compounds attract necrophagous insects and originate from microbes breaking down sulfur amino acids like cysteine and methionine. Alcohols result from bacterial degradation of amino acids, fatty acids, and carbohydrates. Aromatic compounds originate from the metabolism of amino acids, such as tyrosine and tryptophan, while nitrogen compounds are associated with protein breakdown [61].

The dry stage (over 50 days) involves skeletonization and bone diagenesis, which can take centuries for complete disappearance. To sum up, the numerous decay VOCs, they can be grouped based on their likely originating biomolecules: proteins yield alcohols, acids, aromatics, nitrogen, and sulfur compounds; acids produce indolic/phenolic compounds and alcohols; lipids generate alcohols, acids, aldehydes, ketones, and hydrocarbons; and carbohydrates form alcohols, acids, and ketones [61].

Consistent with these stage-specific patterns, Table S1 consolidates recurrent markers across matrices and PMIs: sulfur volatiles dominated by DMDS and DMTS; nitrogen-containing indole, pyridine congeners, and trimethylamine; lipid-oxidation aldehydes from hexanal to decanal; short-chain fatty acids (acetic, propanoic, butanoic); mid-chain ketones (acetone, 2-butanone, 2-heptanone, 2-nonanone); and a reproducible hydrocarbon background from C7–C16 alkanes. Environmental and handling-related volatiles, including halogenated solvents, glycol ethers, and cyclic siloxanes, also frequently appear in indoor or clinical samples and should be treated as confounders (Table S1). Importantly, Table S1 shows that DMDS/DMTS, early lipid aldehydes, and SCFAs co-occur and evolve across stages—DMTS often strengthening relative to DMDS—as moisture levels decrease and microbial communities shift, whereas hydrocarbons and some phenolics persist in skeletonized/bone-dominant contexts (Table S1).

Differences between humans and animal surrogates also emerge in the compiled literature: although pigs are valuable analogs, human datasets in Table S1 more frequently report ester-rich and nitrogen–sulfur signatures in confined headspace (e.g., morgue/body-bag air), while textiles and soils can partition or retain specific classes (e.g., suppression of sulfur volatiles in fabrics noted by [57]) (Table S1). These observations caution against direct transfer of surrogate-derived VOC priors to all human contexts without matrix-specific validation.

### 2.3. Sigma Pseudo™ Corpse Scent: Synthetic Replication of Decomposition

The odor profiles mentioned earlier are crucial in forensic work, especially for training cadaver-detection dogs. However, ethical and logistical issues restrict access to human remains for training, increasing the need for synthetic cadaver scent simulants. Commercial pseudo-cadaver formulations—such as Sigma Pseudo™ Corpse Scent Formulation I (PSI) and Formulation II (PSII), marketed by Sigma-Aldrich^®^, St. Louis, MO, USA, (now MilliporeSigma^®^, Burlington, MA, USA)—purport to replicate key decomposition VOCs for standardized dog training. These proprietary products are marketed as mimicking stage-specific decomposition odors: PSI focuses on early decay, while PSII mimics advanced decay (post-putrification detection). However, independent validation of their chemical accuracy remains limited. Tipple et al. [62] evaluated the composition of PSI and PSII. Six complementary techniques were used to thoroughly profile VOCs, including direct liquid injection (1D GC-MS & 2D GC × GC-TOFMS) and headspace sampling through (a) Solid-Phase Microextraction (SPME), (b) Purge and Trap (P/T), (c) Ambient Preconcentration/Thermal Desorption (ACEM), and (d) Cryogenic Preconcentration/Thermal Desorption analyses. Their results are summarized in Table 1.

Direct liquid injection analysis using 1D GC-MS revealed key compositional differences: PSI mainly consisted of 2-pyrrolidinone (28 ± 4%) and 4-aminobutanoic acid (GABA) (71 ± 5%), with minor traces of methylated pyrrolidinones. In contrast, PSII contained putrescine (11 ± 1%), cadaverine (11 ± 1%), 2-pyrrolidinone (24 ± 5%), and 4-aminobutanoic acid (54 ± 7%). Additionally, comprehensive two-dimensional GC × GC-TOFMS analysis confirmed these major components and identified further trace compounds in these formulations [62]. Similar findings were observed with the use of GC × GC-TOFMS by Stadler et al. [63].

Interestingly, headspace analysis techniques produced different profiles compared to liquid injection. SPME revealed the dominant volatile to be 2-methyl-1-(1,1-dimethylethyl)-2-methyl-1,3-propanediyl propanoic acid (MDMPP), acetone, and 2-ethyl-1-hexanol, but notably failed to detect PSII’s putrescine/cadaverine, despite their volatility. Purge/trap primarily released acetone (93%) from PSI versus a mix of volatiles from PSII. ACEM detected ketones and alcohols but missed key components. Cryogenic analysis identified over 75 volatiles per formulation, mainly acetone and ethanol, rather than decomposition markers. Importantly, less than 25% of the detected headspace compounds matched known decomposition profiles, with major constituents (2-pyrrolidinone, 4-aminobutyric acid) showing negligible volatility [62]. To evaluate the suitability of commercially available pseudo-scents as canine training aids, we assessed their chemical fidelity, defined here as the degree to which synthetic mixtures reproduce key volatile organic compounds (VOCs) consistently detected in human decomposition headspace (Table S1).

The use of these pseudo scents for training cadaver dogs is questioned because their composition, which features only a few compounds such as putrescine, cadaverine, and GABA, greatly oversimplifies the decomposition odor, which involves hundreds of compounds. Importantly, none of the key decomposition volatiles, such as polysulfides, were present, and the specific compounds identified have not been reported in decomposition headspace studies. This mismatch raises concerns about their effectiveness as training aids, especially since dogs trained on real remains do not recognize these pseudo scents [63].

Consistent with decomposition headspace literature, sulfur-containing VOCs—especially dimethyl disulfide (DMDS) and dimethyl trisulfide (DMTS)—are recurrent, highly odor-active markers of human remains [8]. In contrast, across analytical modes (SPME, purge-and-trap, ACEM, cryogenic preconcentration), Sigma Pseudo™ Formulations I/II exhibited negligible or non-detectable sulfur volatiles; <25% of detected headspace compounds overlapped with known decomposition profiles, and major constituents (e.g., 2-pyrrolidinone, 4-aminobutanoic acid) showed minimal volatility [62,63]. This divergence defines a chemical fidelity gap dominated by the absence of sulfur cues and provides a plausible mechanistic basis for reports that dogs trained on authentic biological materials do not generalize to these synthetics [16].

In addition to Sigma Pseudo™ formulations, ScentLogix™ HRD scent kits have gained popularity as synthetic training aids for HRD dogs, offering multi-purpose odor references designed for use across land, air, and water environments. Although peer-reviewed chemical validation is currently limited, the manufacturer states that the kits replicate the odor signatures of two human cadavers, encompass all stages of decomposition, and are intended for canine imprinting and maintenance training programs aligned with international detection and certification standards [64]. When contrasted explicitly with Table S1, the Sigma headspace is dominated by small ketones and alcohols (acetone, isopropanol/ethanol, 2-butanone) with episodic detection of a few aldehydes and ethers, whereas human-remains headspace consistently includes sulfur volatiles (DMDS, DMTS), SCFAs (acetic, propanoic, butanoic acids), lipid-oxidation aldehydes (hexanal–nonanal), nitrogen heterocycles and amines (indole, pyridine, trimethylamine), and mid-chain ketones (2-heptanone, 2-nonanone) (Table S1). Sigma’s liquid-phase “major components” (2-pyrrolidinone, GABA, putrescine, cadaverine) have low headspace contributions under ambient conditions and are either absent or rarely reported in authentic decomposition headspace (Table S1), creating a stage-agnostic odor that does not track the temporal evolution observed in fresh through advanced decay. Moreover, key odor-active sulfur cues central to insect attraction and canine detection are under-represented or absent in Sigma headspace, while environmental confounders common in morgue/body-bag settings (e.g., halogenated solvents, glycol ethers, siloxanes) cannot substitute for microbiologically generated sulfur and acid chemistries (Table S1). Taken together, these differences rationalize performance gaps reported for dogs trained on authentic material versus pseudo-scents [63] and indicate that fidelity-improved training aids should prioritize inclusion (and controlled release) of DMDS/DMTS, early lipid aldehydes, SCFAs, and representative nitrogenous volatiles, tuned to stage-specific emissions profiles (Table S1).

Finally, although ScentLogix™ HRD kits and Sigma Pseudo™ Corpse Scent Formulations I (PSI) and II (PSII) are widely used, independent GC/GC × GC validation remains sparse. Table S1 provides a practical blueprint for benchmarking any commercial mimic against human-specific headspace, including overlap with sulfur volatiles, SCFAs, C6–C10 aldehydes, and mid-chain ketones, which should be quantified alongside the avoidance of other volatiles. Incorporating these criteria would align synthetic aids more closely with the recurrent human-remains odor space.

## 3. Canine Detection of Decomposition Odor

### 3.1. Introduction to the Biological Basis of Canine Olfaction

Over roughly 30,000 years of co-evolution with humans [65], dogs have become skilled at reading human cues [66,67]. However, olfaction is equally crucial in canine cognition and communication [68]. For humans, smell is less vital, though the COVID-19 pandemic highlighted its importance when anosmia significantly disrupted daily life [69].

Dogs, as macrosmatic animals, have highly developed olfactory systems essential to survival, unlike microsmatic humans [70]. Dogs have around 250–300 million receptors across 150–170 cm^2^ of epithelium, compared to humans’ 5 million over 5–6 cm^2^. This allows detection of odorants at concentrations 1000 to 1,000,000 times lower than humans can perceive [70]. The canine OB is also about 40 times larger relative to brain size than the human equivalent [71]. Functionally, dogs discriminate odors with far greater precision and can detect trace levels down to parts per trillion [72], making them unmatched biological detectors.

#### 3.1.1. Genetics of Canine Olfaction

About 80% of canine olfactory receptor (OR) genes are functional versus roughly 50% in humans. OR gene numbers evolved through a “birth-and-death” process and can be shaped by environmental exposure and training. Comparative studies with wolves and coyotes suggest differences stem more from behavior than gene count [73,74].

Dogs possess an exceptionally large repertoire of olfactory receptor (OR) genes, with approximately 1094 functional copies compared to ~400 in humans, providing a strong molecular foundation for their heightened olfactory sensitivity [21,22]. Many of these OR genes are expressed at vastly different levels, with expression ratios exceeding 10,000-fold, revealing a highly tunable and specialized olfactory epithelium [20]. This diversity in both repertoire and expression may contribute to subtle individual and breed-related differences in detection thresholds and odor generalization.

The genetic architecture of canine olfaction further underscores its evolutionary specialization. Olfactory receptor genes are organized in approximately 40 clusters distributed across multiple chromosomes, with major clusters on chromosomes 18 and 21 [22]. Comparative genomics has revealed that dogs have retained one of the largest functional OR repertoires among mammals, despite lineage-specific pseudogenization events, reflecting evolutionary selection for olfactory acuity in hunting and tracking [21]. Breed-related studies suggest that selective breeding has also shaped allelic diversity in OR genes, with certain variants more prevalent in scent-oriented breeds such as retrievers and spaniels [23]. This genetic variability provides a plausible explanation for differences in HRD performance across individual dogs and breeds, complementing the influence of training and environment. Moreover, dynamic regulation of OR gene expression has been observed, with age-related shifts and modulation by environmental factors such as chronic nasal inflammation [20]. Together, these findings highlight that canine scent detection is not only chemically and neurologically mediated, but also deeply rooted in genomic diversity and regulation. Table 2 summarizes the genetic foundations of canine olfaction, highlighting how genomic diversity and receptor specialization underpin the exceptional detection abilities of HRD dogs.

#### 3.1.2. Anatomy of Nasal Cavity and Nasal Turbinates

**Figure 2:**
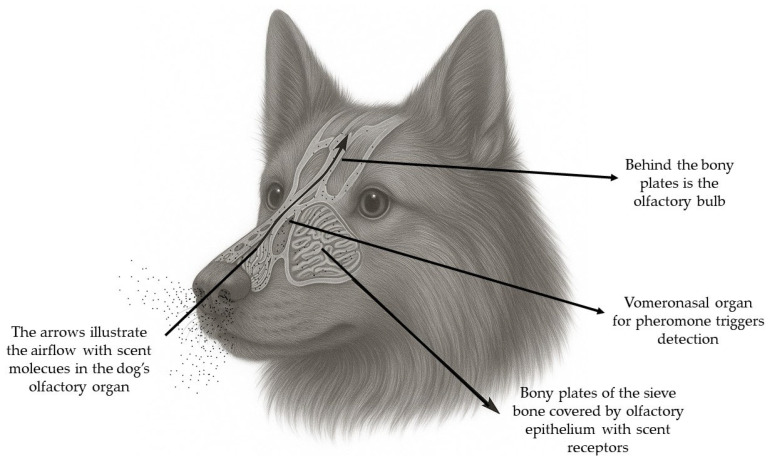
Anatomy and airflow through the dog’s olfactory organ.

The canine nasal cavity contains a highly complex system of thin bony turbinates covered with epithelium, which greatly increase the olfactory surface area and optimize airflow. Around 5–15% of inhaled air reaches the olfactory region, while the rest moves into the respiratory tract. This intricate network directs odorant molecules efficiently toward receptors. The turbinates are lined with specialized olfactory epithelium containing millions of olfactory receptor neurons (ORNs) (Figure 2). Each neuron has hundreds of cilia capable of detecting odors, and with up to 2 billion neurons, dogs achieve exceptional olfactory sensitivity [75].

The epithelium regenerates continuously, as ORNs live only 30–60 days before being replaced by progenitor cells. Neurogenesis of olfactory bulb (OB)-related cells is also influenced by activity [75,76]. Supporting cells and Bowman’s glands produce mucus to maintain hydration and help dissolve odorant molecules [77].

Sniffing is a rapid, repetitive process (4–7 Hz) that increases odor interaction with the olfactory epithelium. During sniffing, about 12–13% of air is directed toward the turbinates [78]. Each nostril works independently, aiding in the localization of odor sources. Different volatile compounds deposit in distinct epithelium regions, improving detection. Sniffing is both mechanical and cognitive dogs adjust frequency for recognition and localization [19,79].

Dogs also have a vomeronasal organ (VNO) located near the nasal septum, which detects non-volatile compounds, such as pheromones, in urine or saliva. VNO information reaches the OB, hypothalamus, and limbic system, influencing sexual and social behavior [19].

Signals from ORNs travel via the olfactory nerve to the OB. Within glomeruli, mitral and projection cells integrate input, modulated by interneurons. Lateral inhibition sharpens recognition, and synchronized OB neural oscillations enhance precision [77]. Advanced imaging has revealed five major OB projection pathways to the frontal lobe, piriform cortex, hippocampus, entorhinal cortex, and occipital lobe, suggesting close olfaction-vision integration [79]. Each ORN expresses only one receptor type; combined receptor activation patterns encode odors [19,75,80].

#### 3.1.3. The Molecular Basis of Signal Transduction

**Figure 3:**
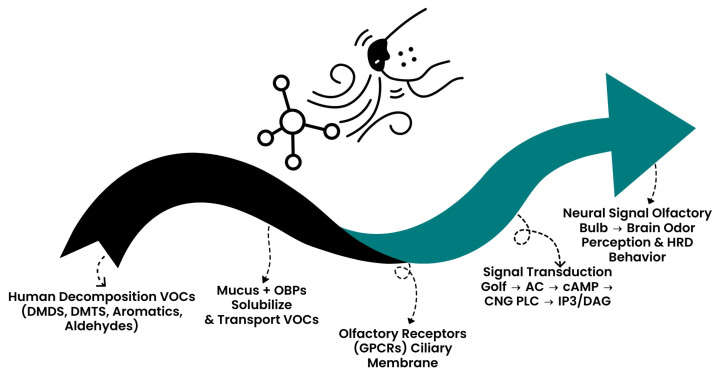
Molecular and membrane-level mechanisms of canine olfaction. Volatile organic compounds (VOCs) such as sulfur-containing molecules and amines dissolve in the nasal mucus, where olfactory binding proteins (OBPs) facilitate their transport to olfactory receptors (ORs) located in the ciliary membrane of sensory neurons. Ligand binding activates intracellular cascades, including the Golf–adenylyl cyclase–cAMP–CNG channel pathway and the PLC–IP3/DAG pathway, resulting in neuronal depolarization. Action potentials are transmitted to the olfactory bulb, where odor maps are formed, providing the molecular and neurobiological basis for the exceptional scent detection abilities of HRD dogs.

Odorant detection does not occur in isolation at the receptor level but is strongly shaped by the surrounding membrane environment. Olfactory receptor proteins are embedded in the ciliary membrane of sensory neurons, where odorants must first be solubilized by olfactory binding proteins (OBPs) secreted by Bowman’s glands in the nasal mucus [81]. This step facilitates transport of hydrophobic VOCs, including many decomposition products, toward the receptor surface. Signal transduction relies on two parallel intracellular cascades: the canonical Golf–adenylyl cyclase–cAMP–CNG channel pathway and a phospholipase C–IP3/DAG pathway, both converging on membrane depolarization [23]. Importantly, receptor activation is dynamically regulated by feedback mechanisms such as PDE-mediated cAMP degradation and Ca^2+^/calmodulin modulation of CNG channels, which underlie olfactory adaptation to persistent odors. Furthermore, the olfactory epithelium demonstrates remarkable neurogenesis, with basal cells continuously regenerating into new sensory neurons—a unique case of neuronal replacement in the mammalian nervous system [82]. These membrane- and mucus-level processes ensure both sensitivity to trace VOCs and resilience of the canine olfactory system in long-term detection work. These processes are summarized in Figure 3, which illustrates how volatile compounds travel through the mucus and olfactory binding proteins, interact with receptors in the ciliary membrane, and trigger signal transduction pathways that ultimately generate odor perception in the canine brain.

**Figure 4:**
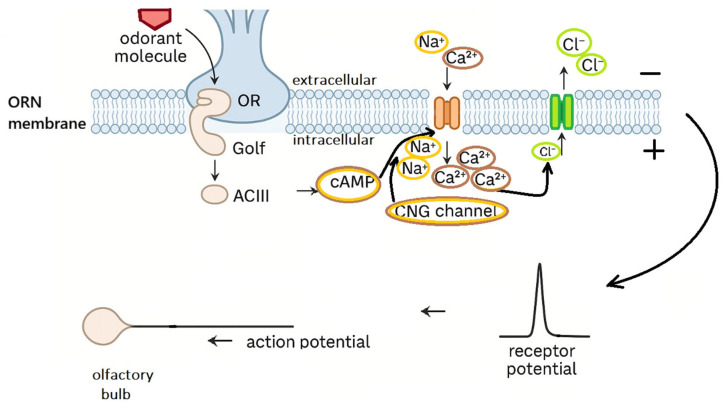
Graphic of the ORN signaling cascade triggered by odorant–receptor binding.

The process of odor detection begins in the cilia of olfactory receptor neurons (ORNs) within the olfactory epithelium. An odorant molecule binds to a G protein-coupled receptor (OR), leading to the activation of the Golf protein. The activated Golf protein stimulates adenylyl cyclase type III (ACIII), resulting in increased intracellular levels of cyclic AMP (cAMP). Elevated cAMP opens cyclic nucleotide-gated (CNG) cation channels, allowing the influx of Na^+^ and Ca^2+^ ions. The rise in intracellular Ca^2+^ subsequently activates Ca^2+^-dependent chloride channels, amplifying the depolarization of the membrane. If the receptor potential reaches threshold, an action potential is generated and transmitted along the axon of the ORN to the olfactory bulb. (Figure 4).

Molecule -receptor interaction follows a docking theory where odorant molecular shape and non-covalent interactions, potentially including metal coordination, govern OR activation [83].

Direct recording of action potentials from single ORNs in dogs remains technically challenging. In practice, indirect methods such as electro-olfactography (EOG), functional magnetic resonance imaging (fMRI), and olfactory-targeted electroencephalography are employed. Myers and colleagues [84] demonstrated that EOG is an effective technique for evaluating the functional capacity of the canine olfactory epithelium, enabling the measurement of summed generator potentials in response to odorants. More recent work, including that of Jia et al. [85], employed fMRI to visualize brain activity in conscious dogs in response to olfactory stimuli, confirming activation of both the olfactory bulb and cortical regions.

An intriguing line of research concerns the modulation of neuronal excitation strength. Ramaihgari et al. [75] reported that the addition of zinc nanoparticles can increase OB and hippocampal activity and boost neuronal connectivity two- to threefold [75,80]. In a subsequent study, Jia et al. [85] confirmed this using fMRI, showing that olfactory signals were stronger and more widespread following Zn-NP exposure. These findings suggest that chemical modulators can increase the sensitivity of canine ORNs, with potential applications in enhancing the performance of detection dogs.

Importantly, low-molecular-weight sulfur compounds, such as thiols and sulfides, are known to have highly potent odors even at extremely low concentrations, and are critical components of decomposition VOCs [8]. The molecular detection of such compounds may involve ORs functioning as metalloproteins, with copper or zinc ions facilitating binding—evidence supports the essential role of metals in enhancing OR responses to sulfurous odorants [27].

Clinical investigations demonstrate that EOG can also be applied in the diagnosis of conditions leading to anosmia. Myers and colleagues [86] described the loss of olfactory epithelium function in dogs with distemper, in which recorded potentials were significantly diminished. More recent reports [87] indicate that the method may be useful in assessing olfaction in dogs with sudden acquired retinal degeneration syndrome (SARDS), enabling an objective evaluation of sensory impairment.

Studies by Grosmaitre et al. [88] and Ghatpande and Reisert [89] revealed that action potentials may be generated at the level of ORN dendritic knobs, enabling rapid and precise responses to dynamic odor stimuli. The application of patch-clamp and loose-patch techniques provided deeper insights into the dynamic processes underlying odor coding. These findings indicate that although the molecular mechanism of olfactory transduction is conserved across species, differences in epithelial organization and ORN numbers shape species-specific olfactory sensitivity.

Once an action potential is generated in an ORN, the signal reaches the olfactory bulb, where synaptic transmission to mitral and tufted cells enables further processing. Margrie et al. [90] demonstrated that action potentials propagate along mitral cell dendrites, allowing broad synchronization of output signals. Schoppa and Westbrook [91] documented that inhibitory interneurons, particularly granule cells, regulate oscillatory firing patterns through lateral inhibition, ensuring precision in odor coding. Studies by Kashiwadani et al. [92] further highlighted the importance of network oscillations in odor discrimination.

Understanding the excitation of ORNs and the generation of action potentials has significant practical value. In dogs trained for detection tasks—such as identifying narcotics, explosives, or cadaver scent olfactory sensitivity could improve performance.

Connections from the OB to the hippocampus and amygdala contribute to emotional responses, memory links, and episodic recall triggered by odors. OB activity can synchronize with other brain regions during sniffing [70]. Dogs excel in detecting explosives, narcotics, diseases, and locating people or remains. Their detection threshold often surpasses that of laboratory instruments, thanks to sniffing efficiency and advanced OB processing.

Odor detection in dogs and other macrosmatic species is based on conserved molecular mechanisms. Although research in dogs is mainly limited to indirect methods such as EOG and fMRI, rodent models provide clear electrophysiological evidence that ORN activation leads to action potential generation and subsequent signal transmission to higher levels of the olfactory pathway. Integrating these findings provides a comprehensive understanding of the exceptional sensitivity of canine olfaction and the mechanisms that can be modulated for both practical and clinical applications.

Some ORs may also show enhanced sensitivity to sulfur-containing volatiles through metal ion cofactors in the binding pocket, which explains the extremely low detection thresholds of thiols and polysulfides associated with human decomposition [26,27]. This molecular perspective directly links decomposition chemistry with canine perception and sets the stage for the sulfur gap hypothesis discussed later.

In addition to classical ORs, trace amine-associated receptors (TAARs) play a specialized role in detecting volatile amines such as trimethylamine, putrescine, and cadaverine. These compounds are strongly associated with decomposition and “danger/decay” signals in vertebrates [24,25]. The presence of such amines in decomposition VOC profiles suggests that TAAR activation may also contribute to the unique cadaveric odor signature, reinforcing the importance of including amine components in synthetic training mixtures and potentially explaining inter-individual variation in canine behavioral responses.

### 3.2. HRD Dog Training and Scent Detection

HRD dogs are specially trained to detect a wide range of odors associated with the decomposition of human tissues—from fresh remains through advanced decomposition stages to odors associated with skeletal remains and bodily fluids. The literature distinguishes four primary categories: fresh matter, decomposition fluids, volatile organic compounds (VOCs) formed during advanced decay, and odors related to bones and residual minerals. These dogs search for these odors in various environments, both open and enclosed, including rubble, avalanches, and underwater [3].

Dargan et al. [93,94] analyzed volatile compounds emitted from ethically sourced training materials—amputated human tissues obtained from hospitals. Alongside volatile organic compounds (VOCs), sevoflurane, a volatile anesthetic used during surgical procedures—was consistently detected, representing a notable extraneous signal relative to the odor profile typical of decomposition. This observation indicates that ethically sourced clinical materials may retain procedural artifacts that influence their chemical signature.

Regarding VOC profile stability, older (“matured”) specimens exhibited lower temporal variability than freshly obtained samples, suggesting a progressive stabilization of the odor profile over time. Notably, variations in storage conditions did not produce significant differences in the overall VOC composition of the analyzed samples.

Environmental parameters such as temperature, wind, humidity, and substrate type exert a significant influence on the dispersion dynamics of volatile organic compounds (VOCs) in the environment, encompassing both synthetic odorants employed in canine training protocols and naturally occurring odor sources. Empirical studies indicate that elevated temperatures and higher humidity levels generally facilitate the atmospheric dissemination of odorant molecules, whereas pronounced thermal fluctuations or arid conditions may accelerate molecular degradation or promote rapid dilution. Likewise, substrates with low porosity, such as compacted or impermeable surfaces, as well as dense vegetative cover, can impede molecular diffusion, thereby constraining the free spatial migration of VOCs and limiting their localized concentration [3].

The training paradigm facilitates the canine’s capacity to discriminate the designated target odor from both background olfactory stimuli and environmental scent contaminants, encompassing, among other things, chemical agents employed during amputation procedures of ethically sourced training materials. Beyond anesthetic compounds, such extraneous odorants include the immediate microenvironment of the material, such as packaging substrates, detergents, and absorbent matrices, which may contribute extrinsic volatile organic compounds (VOCs). Accordingly, training protocols should be meticulously designed to mitigate the inadvertent conditioning of the subject to background odor profiles. Within the proficiency consolidation phase, olfactory generalization may ensue, thereby extending detection competencies to novel material specimens presented for pre-search scent imprinting [93,94,95,96].

Selection and training methods vary between agencies and countries, with a general lack of standardized, evidence-based guidelines. There is no uniform standard by which HRD teams are trained [96].

The training of scent detection teams serving humans is similar regardless of the scent being sought. It begins with familiarizing the handlers with regulations, dog psychology, and training methods. The dog is initially socialized, taught obedience, and later taught to distinguish between scents, which become increasingly complex and diverse over time, in increasingly complex environments, leading to the generalization of the dog’s response to scent mixtures containing the learned component and the consolidation of detection accuracy.

Sidel et al. [96] identified key behavioral and physiological indicators of emotional state, such as heart rate variability (HRV) and quantitative behavioral assessment (QBA), in working dogs. Martin et al. [95] analyzed working dogs (including HRD) and laid the foundation for a five-domain model of welfare in HRD training programs. Recommendations included: adequate rest periods; regular veterinary examinations; scheduled breaks during periods of high workload; physiological and behavioral monitoring; and training handlers to detect signs of fatigue and stress.

Synthetically produced versions components of the smell of decay are controlled, safe training materials for HRD dogs. Synthetic scents allow precise composition control, reducing variability found in natural sources. They minimize biological risk and legal complications when working with human materials. However, limitations exist: synthetics may not replicate the full complexity of VOC profiles found in real remains (e.g., amines, aldehydes, ketones, alcohols, disulfides), which can affect real-world detection by dogs trained exclusively on synthetics [63,97].

#### Examples of Sigma Use in Training Programs

FEMA (Federal Emergency Management Agency), under the Urban Search and Rescue program, maintained 90 task forces in 2020, many with certified cadaver dogs. These dogs are deployed to locate disaster victims, both live (Life Finding Dogs) and deceased—in urban and post-disaster environments like rubble, ruins, fires, and collapsed buildings. While FEMA does not disclose detailed training protocols, it stresses the importance of “controlled environment scent training” using synthetic odors. Some FEMA dogs have dual certification (LFD and HRD), especially in military or specialist teams. FEMA HRD dogs were deployed after the 9/11 attacks, hurricanes Katrina and Harvey, earthquakes, and the Minneapolis bridge collapse (2007). FEMA provides training guidelines and assessment protocols for canine performance under high-stress conditions [98].

Elite K9 (Connecticut) has used Sigma Formulations for years in HRD training. Their varied training scenarios allow comprehensive skill development. Elite K9 employs two Sigma formulas: (a) Formula I: high-concentration sample for initial scent-reward association; effective in cold environments, and (b) Formula II: adjustable dilution for advanced scenarios (e.g., buried, submerged, elevated hides, chemical/food distractors, unknown-to-handler setups). Formulas are packaged in single-use vials, applied to gauze in plastic scent tubes, and can be buried, suspended, or used as toys. Dogs show varying reactions (from full motivation to cautious interest), allowing precise training adjustments and positive reinforcement [99]. Federal agencies such as NOAA and FEMA evaluate synthetics as supplements to real samples, supported by DHS reports and training videos, indicating growing Sigma Formulations use in operational testing (e.g., extreme temperatures) [100]. In Table 3 summarizes the documented use of Sigma pseudo-scents simulating the odor of decomposing human remains for training working dogs across countries.

### 3.3. Performance and Limitations

#### 3.3.1. Sensitivity, Specificity, and False Alerts

The performance of human-remains detection (HRD/CDD) canines is primarily evaluated through sensitivity, specificity, and rate of false alerts. Sensitivity refers to the dog’s ability to correctly indicate the presence of a target odor, while specificity denotes its capacity to correctly disregard irrelevant odors. These metrics are critical for the reliability and utility of detection dogs in forensic and rescue operations.

In one study, cadaver dogs trained to detect human-remains odors on contaminated surfaces achieved sensitivity levels of 75–100% and specificity of 91–100%, resulting in overall accuracy of 92–100%, even up to 65 days after the sample was exposed to odor sources [96]. False alert rates tend to be higher during the initial training phase, especially when encountering novel conditions or distractors; however, after just a few subsequent sessions, detection accuracy approaches 100%, with false alerts dropping close to zero [95]. According to DeGreeff [3], training with actual human tissue results in approximately 90% correct responses and minimizes false alerts.

At the molecular level, sensitivity and specificity can also be shaped by polymorphisms in OR genes. Certain allelic variants have been correlated with differences in detection outcomes in working dogs [23]. Alongside imprinting history and environmental influences, genetic variability in OR repertoires may therefore contribute to individual thresholds of detection, susceptibility to false alerts, and long-term stability of odor memory [21,22]. In practical training contexts, this underlines the need for individualized protocols and validation tests.

#### 3.3.2. Environmental and Operational Challenges

Dargan and Forbes [94] highlighted that human remains and decomposition fluids emit rich VOC profiles (acids, ketones, sulfur compounds) that facilitate detection, but factors such as soil type (clay vs. sand), humidity, and temperature significantly affect VOC diffusion and signal persistence. Alexander et al. [105] found that in sand, dogs exhibited significantly shorter detection times than in clay soils (*p* < 0.001), indicating that sandy substrates permit faster odor release. Despite these temporal differences, dogs achieved comparable detection accuracy across both soil types.

#### 3.3.3. Legal and Ethical Considerations in Canine Evidence

Ensminger et al. [106] emphasized that canine scent identification is a valuable tool in criminal proceedings—particularly when conventional visual identification is hindered by lack of witnesses, time elapsed, or body condition. However, courts may admit scent-based evidence even when protocols are flawed, establishing reliability through precedent, which can undermine its credibility. Ensminger et al. [106] proposed stricter standards for scent evidence, recommending: pretrial training and testing with a diagnostic odds ratio of at least 10:1 (target hits vs. false alerts); observer-blind testing to eliminate handler bias; confirmation of scent indication by a minimum of two, ideally three, dogs to bolster evidentiary strength; corroboration of scent evidence by independent sources; ongoing research and collaboration with the scientific community to maintain standardization and legal credibility.

## 4. Linking Chemistry and Canine Detection: Sigma as a Training Tool

### 4.1. How Sigma’s Chemical Profile Matches Canine Detection Capabilities

The olfactory perceptual abilities of dogs encompass an exceptionally broad spectrum of chemical compounds, rendering them highly effective tools in scent detection tasks. Dogs can be successfully trained to recognize and signal the presence of nearly any odor, whether natural or synthetic, provided that the chemical composition of the target odor is known and the substance is either replicable or obtainable for training purposes.

A major limitation in the training process lies in the availability and chemical characterization of the odorant compound targeted for detection. In cases where odors are unknown, chemically unstable, or difficult to access, such as those associated with the decomposition of human remains—training opportunities may be significantly restricted.

In recent years, the rapid advancement of analytical techniques, particularly gas chromatography–mass spectrometry (GC-MS), has enabled precise identification and characterization of volatile organic compounds (VOCs) emitted by a wide range of biological and chemical materials. These developments have made it possible to design and synthesize more accurate, controlled, and safe odor substitutes, which increasingly replicate the complexity and scent profile of original biological sources, such as human decomposition.

Human decomposition produces a wide spectrum of volatile organic compounds (VOCs), including amines, short-chain fatty acids, indoles, phenols, esters, aldehydes, and, importantly, sulfur-containing compounds. Biogenic diamines such as cadaverine and putrescine can be abundant in tissues and fluids, but in headspace they are often less prominent than low–molecular-weight amines (e.g., trimethylamine), *N*-heterocycles (e.g., indole), and phenols (e.g., *p*-cresol). Aldehydes—particularly C6–C9 n-alkanals (hexanal–nonanal)—reflect lipid peroxidation, whereas esters and SCFAs reflect lipid hydrolysis and microbial fermentation. Among all classes, volatile sulfur compounds—particularly dimethyl sulfide (DMS), dimethyl disulfide (DMDS), and dimethyl trisulfide (DMTS)—are exceptionally odor-active at trace levels and are consistently reported in decomposition headspace across matrices (Table S1) [8], with DMTS often strengthening relative to DMDS as decomposition progresses. These cues are widely regarded as critical to the unique olfactory profile of human remains and to reliable detection by cadaver dogs. Building on this chemical background, we next evaluate how current synthetic products—particularly the Sigma formulations—compare in chemical fidelity to authentic human headspace and how such differences may translate to canine responses. As a result, modern training aids with high molecular fidelity to authentic samples are now being developed. These can be utilized in both laboratory and field settings, offering biological safety and enhanced standardization of canine training protocols.

### 4.2. Current Evidence on Canine Responses to Sigma vs. Real Human Remains

In the study by Caldwell et al. [16], an experiment was conducted using three types of Sigma synthetic odorants: Pseudo™ Corpse Scent Formulation I, Pseudo™ Corpse Scent Formulation II, and Pseudo™ Corpse Scent Drowned Victim. The dogs selected for the study had previously located human remains under natural field conditions and had been trained exclusively with realistic biological materials (e.g., tissues, fragments of actual human remains). The detectability of these synthetic odorants was tested under both laboratory and field conditions. None of the tested dogs showed any indication or behavioral response to the synthetic scents, either in indoor or outdoor settings. The authors concluded that the synthetic compounds were chemically too simplistic and lacked key volatile organic compounds (VOCs) necessary for the dogs to classify them as cadaveric. The dogs, having been trained on authentic biological samples, did not generalize their detection behavior to the synthetic alternatives [16].

In a more recent study by Martin et al. [2], the researchers analyzed the olfactory responses of four certified Human Remains Detection (HRD) dogs, all English Springer Spaniels, to varying concentrations of specific compounds typically released during human decomposition. Similarly to the earlier study, all dogs had been trained exclusively using biological materials, and testing was conducted in both controlled laboratory and natural outdoor environments. The initial synthetic blend tested consisted of dimethyldisulfide (DMDS), diethyl disulfide (DEDS), pyrrole, *p*-cresol, and indole.

Two out of the four dogs clearly recognized and alerted to the initial mixture, indicating detection of a cadaveric odor. A third dog showed hesitation, sniffing the sample for more than four seconds without a definitive response, and the fourth dog did not react at all. Notably, the dogs maintained consistent positive responses to the blend in both environments even at a dilution of 1:1000 (10^−3^), suggesting that the scent remained behaviorally effective even at very low concentrations.

Moreover, the dogs continued to alert to the scent even when a single compound was removed from the mixture, indicating a degree of perceptual robustness. These findings suggest that the sulfur- and nitrogen-containing compounds are likely the most behaviorally relevant cues in cadaveric scent detection. When the mixture contained only plant-based odorants without any cadaveric components, the dogs withheld responses, but the addition of a single cadaveric molecule was sufficient to reinstate alerting behavior. Despite undergoing the same training regimen, each dog responded slightly differently to variations in mixture composition, highlighting individual olfactory perception differences.

These results underscore the growing potential of synthetic odorants in forensic canine training. Over time, increasingly refined and chemically controlled synthetic training aids are being developed. These formulations are easier to produce, transport, and standardize than biological materials, yet they are still capable of eliciting reliable detection behavior in trained dogs [2]. While these behavioral findings highlight both the promise and the limitations of synthetic mixtures, a deeper understanding of canine olfaction at the molecular level is needed to explain why certain compounds succeed or fail in training contexts.

### 4.3. Molecular Mechanisms of Canine Olfaction as a Framework for Designing Synthetic Training Aids

The molecular basis of canine olfaction provides a crucial bridge between chemical composition of decomposition odors and their biological perception by dogs. Understanding how volatile organic compounds (VOCs) interact with olfactory receptors (ORs), binding proteins, and downstream signaling cascades is essential for designing synthetic training aids that reliably mimic cadaver scent. Rather than focusing solely on the presence or absence of certain compounds, a receptor-level perspective highlights which molecules are most likely to activate biologically relevant pathways in the canine nose and therefore drive effective odor imprinting.

Olfactory receptors (ORs) are G-protein–coupled receptors that transduce VOC binding into neural signals via canonical Golf–adenylyl cyclase–cAMP pathways [22]. Among these ligands, low-molecular-weight sulfur compounds such as thiols and sulfides are exceptionally odor-active and represent critical markers of decomposition [18]. Evidence further suggests that ORs may function as metalloproteins, with copper or zinc ions enhancing receptor sensitivity specifically to sulfurous odorants [23].

This molecular understanding provides a mechanistic explanation for the limitations of current synthetic training aids. Specifically, our analysis shows that products like Sigma Pseudo™ lack key sulfur-containing VOCs—e.g., dimethyl disulfide (DMDS), dimethyl trisulfide (DMTS), and polysulfides—that are prevalent in genuine decomposition profiles and likely essential for activating specific canine ORs tuned to cadaveric odor signatures. Their absence may lead to incomplete receptor activation and potentially to maladaptive olfactory learning pathways in dogs.

Therefore, we propose an innovative, receptor-guided design approach for synthetic training aids: (1) identification of key VOC agonists for canine ORs, through methods such as in silico docking or in vitro assays using heterologously expressed ORs; (2) formulation of synthetic mixtures that include these high-affinity VOCs—especially sulfur compounds—to ensure activation of the appropriate receptor repertoire; (3) Behavioral validation through structured trials with detection dogs to confirm effectiveness.

This receptor-level framework bridges detailed chemical analysis with canine neurobiology and provides a biologically informed strategy for developing standardized, reproducible, and functionally relevant synthetic training aids. Building on this receptor-level perspective, our chemical analyses point to a critical limitation in current synthetic aids—the systematic absence of sulfur-containing VOCs—which we define as the sulfur gap and elaborate below.

### 4.4. Sulfur Gap-Hypothesis

We propose the “sulfur gap hypothesis”, which suggests that the absence of key sulfur-containing VOCs in current synthetic formulations prevents reliable odor imprinting in cadaver detection dogs. Compounds such as dimethyl disulfide (DMDS) and dimethyl trisulfide (DMTS) are consistently produced during human decomposition and are among the most odor-active markers of the cadaveric odor profile [8]. Their omission in synthetic products such as Sigma Pseudo™ likely results in incomplete activation of the canine olfactory receptor repertoire, particularly receptors tuned to low-molecular-weight sulfides [27]. From a mechanistic perspective, the lack of these compounds may hinder long-term olfactory memory formation, as repeated exposure to incomplete mixtures risks reinforcing irrelevant receptor pathways rather than the biologically relevant cadaver scent image [20,27]. Addressing this sulfur gap should therefore be a priority in receptor-guided design of synthetic aids, ensuring that training materials include the critical VOCs necessary for both chemical and biological fidelity. Recognizing and addressing this sulfur gap not only advances our theoretical understanding of canine olfaction, but also has direct implications for how synthetic aids are formulated, validated, and implemented in forensic training practice.

Temporal dynamics of decomposition VOCs provide an additional dimension of complexity that synthetic aids must capture. Studies using longitudinal sampling have shown that sulfur-containing compounds such as dimethyl disulfide (DMDS) and dimethyl trisulfide (DMTS) dominate during the early stages of decomposition (fresh and bloat), whereas nitrogen-containing amines and indolic compounds increase in prevalence during active and advanced decay [18,63]. Esters and ketones remain prominent throughout decomposition, but their relative abundance is strongly influenced by environmental conditions such as temperature, soil chemistry, and humidity. These stage-dependent shifts suggest that a single, static synthetic mixture is unlikely to reproduce the evolving cadaveric odor landscape. Instead, effective training aids should reflect stage-relevant VOC profiles to ensure that dogs are exposed to the breadth of olfactory cues encountered in operational contexts.

Another key limitation of current synthetic training aids lies in their validation, which has rarely been conducted under operational conditions. Forensic deployments often occur in odor-rich environments where animal remains, decaying vegetation, and anthropogenic chemicals can mask or mimic human decomposition odor. Without systematic field testing across diverse environments—including buried, submerged, or entombed remains—the reliability of synthetic formulations remains uncertain. Beyond field validation, future receptor-guided design could benefit from molecular assays and computational tools, including in vitro testing of heterologously expressed canine olfactory receptors and in silico docking models to predict high-affinity odorant–receptor interactions [20,26]. These approaches would allow rational selection of VOCs most likely to activate biologically relevant receptors, thereby bridging analytical chemistry with canine neurobiology in a way that extends far beyond previous work.

**Figure 5:**
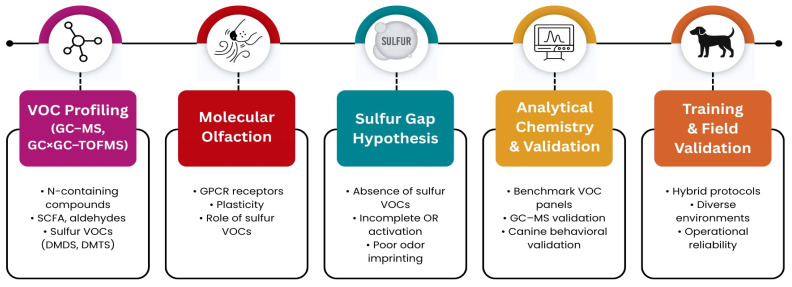
Integrated framework for receptor-guided design of synthetic training aids.

This integrated framework is summarized in Figure 5, which illustrates how chemical profiling, molecular olfaction, and the sulfur gap hypothesis converge to guide analytical validation and practical training strategies.

### 4.5. Importance of Analytical Chemistry in Developing and Validating Synthetic Scents

Analytical chemistry is indispensable for creating scientifically credible synthetic decomposition scents. Its role encompasses three critical phases: identification, replication, and validation. First, advanced techniques such as Gas Chromatography-Mass Spectrometry (GC-MS) and comprehensive two-dimensional gas chromatography (GC × GC-TOFMS) are used to identify and quantify the volatile organic compounds (VOCs) released during human decomposition across various stages (from early putrefaction to skeletonization) and under different environmental conditions. Many studies cited above [2,62,63] established core VOC profiles, including hydrocarbons, halogens, nitrogen- and sulfur-containing compounds, metabolites of proteins, lipids, and carbohydrates. Thanks to this, analytical chemistry allows for accurate replication of these profiles. Moreover, in lab-controlled glass jars simulating decomposition over six months, Rosier et al. [18] compared VOC profiles from human and animal tissues. Sulfur-containing compounds dominated the early stages (fresh and bloat), especially in the first month. As decomposition moved into active and advanced decay stages, nitrogen-containing compounds became more common across all species. Esters and ketones also remained major contributors throughout the process. The study identified eight VOCs (3-methylthio-1-propanol, methyl(methylthio) ethyl disulfide, diethyl disulfide, pyridine, ethyl propionate, propyl propionate, propyl butyrate, ethyl pentanoate) that collectively distinguished human and pig remains from other animals, though not exclusively human. Pyridine was often detected in humans but rarely in other species. Five specific esters (3-methylbutyl pentanoate, 3-methylbutyl 3-methylbutyrate, 3-methylbutyl 2-methylbutyrate, butyl pentanoate, propyl hexanoate) showed potential to separate pig from human remains, especially early on. Limitations include using tissue samples instead of whole bodies and the lack of environmental variables [18]. Understanding these VOC profiles and their evolution through decay stages (early soft tissue to skeletonization) is vital for developing detection tools and standardizing human remains detection canine training [35].

Analyzing data directly improves formulation accuracy, allowing synthetic scents like Sigma Pseudo™ to mimic target compounds, including their ratios, release times, and concentration thresholds across decomposition stages. This stage requires systematic fine-tuning to replicate complex VOC interactions with environmental factors such as soil chemistry, tissue variation, or water systems, which influence odor spread and durability.

Validation occurs through two main methods: instrumental validation with GC-MS, confirming that synthetic blends match natural VOC structures and proportions, and biological validation with controlled canine trials, ensuring scents provoke the same behavioral responses to genuine sources when key chemical profiles are maintained. Moreover, analytical chemistry is essential for ongoing quality assurance, maintaining batch consistency through standardized metrics. This guarantees scent reliability during training and operational use, supporting forensic credibility. Translating these laboratory-based insights into real-world forensic and disaster scenarios, however, requires systematic field validation under operational conditions.

### 4.6. Future Directions for Research and Validation Under Operational Conditions

Despite progress in creating synthetic scents, significant gaps in knowledge still exist regarding how laboratory-developed formulations perform in real-world forensic and disaster response scenarios. Future research should prioritize field validation under operational conditions to confirm reliability across diverse environments. This includes studying how environmental factors, such as temperature variations, humidity, soil types, aquatic systems, and microbial communities, affect the release, dispersal, and durability of synthetic VOCs compared to natural decomposition. Experiments should mimic buried, submerged, or entombed remains to understand scent masking or breakdown pathways. Additionally, long-term evaluations of canine performance with synthetic-only training, across settings like urban rubble, wilderness, or mass-casualty sites, are essential. Standardized metrics for false positives/negatives, handler biases, and odor memory retention over years are needed. Research must also explore how environmental contaminants—such as animal remains, industrial chemicals, or decaying vegetation—interfere with synthetic scent detection. Integrating portable analytical tools like GC-MS or electronic noses with canine teams for real-time verification can create hybrid biological-instrumental systems. Finally, collaboration among chemists, forensic anthropologists, canine ethologists, and field practitioners is vital to bridge the gap between lab research and practical application.

Together, these findings emphasize that advancing cadaver dog training requires an integrated framework that links detailed chemical profiling with molecular olfactory mechanisms, addresses the critical sulfur gap, and applies this knowledge to the design and field validation of synthetic aids.

## 5. Conclusions

Synthetic molecular mimics of human decomposition odor, such as Sigma Pseudo™ Corpse Scent formulations, represent a valuable step toward the safer, standardized, and ethically responsible training of human remains detection (HRD) and search-and-rescue (SAR) dogs. Advances in analytical chemistry, particularly GC-MS and GC × GC-TOFMS, have enabled precise characterization of volatile organic compounds (VOCs) released during human decomposition, forming the scientific foundation for creating synthetic scent substitutes. However, current synthetic products capture only a fraction of the complex VOC profile associated with real decomposition, and significant chemical discrepancies remain. In particular, sulfur-containing volatiles such as dimethyl disulfide (DMDS) and dimethyl trisulfide (DMTS) are absent from most formulations, leading to what we define as the “sulfur gap”, a major limitation in their biological fidelity.

Our work contributes two key innovations. First, we propose benchmark VOC panels—including sulfur-, nitrogen-, and indole-based compounds—as chemical standards for validating synthetic aids. Second, we introduce a receptor-guided design framework that emphasizes the need to target canine olfactory receptor activation rather than simply reproducing chemical similarity. This approach provides a mechanistic rationale for why incomplete synthetic mixtures fail and offers a roadmap for rational improvement. As summarized in Figure 5, this integrated framework links chemical profiling, molecular olfaction, and the sulfur gap hypothesis to guide analytical validation and practical training strategies.

Based on these insights, we recommend a hybrid training model: initial imprinting with improved synthetic aids enriched in biologically relevant VOCs, followed by reinforcement with ethically sourced biological materials and validation under operational conditions. Such a strategy balances ethical constraints with functional reliability, ensuring that training outcomes transfer to real forensic deployments.

In conclusion, by bridging chemistry and canine olfaction, our study provides a new paradigm for the rational design of synthetic cadaver dog training aids. While current synthetic scents cannot yet fully replace human remains, they represent an essential complementary tool, and continued interdisciplinary collaboration will accelerate progress toward highly effective, standardized, and ethical alternatives in HRD and SAR dog training.

## Figures and Tables

**Table 1:** Chemical Characterization of Sigma Pseudo™ Corpse Scent Formulations I (PSI) and II (PSII) determined by multiple analytical techniques. Data derived from direct liquid injection GC-MS, headspace sampling (SPME, Purge & Trap, ACEM, Cryogenic Preconcentration) [62]. Major components (>1% abundance) and key volatile organic compounds (VOCs) are reported.

Parameter	Sigma Pseudo™ Corpse Scent Formulation I	Sigma Pseudo™ Corpse Scent Formulation II
Direct Liquid GC-MS Analysis		
Primary Components	2-Pyrrolidinone (28 ± 4%), 4-Aminobutanoic acid (71 ± 5%)	Putrescine (11 ± 1%), Cadaverine (11 ± 1%), 2-Pyrrolidinone (24 ± 5%), 4-Aminobutanoic acid (54 ± 7%)
Minor Components	3-Methyl-2-pyrrolidinone, 4-Methyl-2-pyrrolidinone (≤1%)	3-Methyl-2-pyrrolidinone, 4-Methyl-2-pyrrolidinone, trace 5-amino-pentanol, ethanol, butyrolactone, acetone, methanol, 1,4-dioxane
Solid phase microextraction (SPME)		
Compounds observed	Acetone, 2-Butanone, 1-Butanol, Heptane, 2,4-dimethyl-furan, Methyl isobutyl ketone, Octane, Methoxy-phenyl-oxime, Heptanal, 2-Butoxy-ethanol, Phenol, 2,4,6-Trimethyl-pyridine, Benzothiazole, 1,3-Bis(1,1-dimethylethyl)-benzene, Tetradecane, 2-Methyl-,1(1,1-dimethylethyl)-2-methyl-1,3-propanediyl propanoic acid	Acetone, 1,4-Dioxane, 2,4-dimethyl-furan, 1-Pentanol, 2-Heptanone, Phenol, 2,4,6-Trimethyl-pyridine, Octanal, 2-Ethyl-1-hexanol, 4-Ethyl-1,3-benzenediol, 2-Nonanone, 2-Decanone, Benzothiazole, 2-Methyl-,1(1,1-dimethylethyl)-2-methyl-1,3-propanediyl propanoic acid, Benzophenones, 24-Bis(1,1-dimethylethyl)-phenol
Purge and trap (P/T) analysis		
Major compounds detected	Acetone (93 ± 3%), isopropanol (1.7 ± 0.1%), 2-butanone (1.3 ± 0.2%)	Acetone (19 ± 2%), 1-vinyl aziridine (28 ± 6%), 1,4-dioxane (14 ± 3%), 1-pentanol (28 ± 10%)
Automated Chemical Environment Monitor (ACEM) headspace analysis		
Key component detected	Acetone	Acetone
Other compounds	α-methylstyrene, acetophenone, nonadecane, phthalate, 2-butanone, 2-pentanone, 3-methyl-2-pentanone, heptanal, and 2-butoxyethanol	2-butanone, 1-butanol, 1,4-dioxane, 1-pentanol, 2,3,4,5-tetrahydropyridazine, α-methylstyrene, acetophenone, diphenyl sulfone, phthalate, 3-methyl-2-pentanone and 2-cyclopenten-1-one
Cryogenic preconcentration analysis		
Major compounds detected	Acetone and isopropanol (86%)	Acetone and ethanol (62%)
Other compounds	Ethanol (4.5%), 1,1-difluoroethane (2.9%), octane (1.2%), and 78 trace compounds (<1%)	1,4-dioxane (4.4%), pentyl formate, (3.6%), 1-bromo-2-propanol (2.7%), 2-butanone (2.2%), 3,4-dimethyldihydrofuran-2,5-dione (1.2%), acetaldehyde (1.1%), 2-amino-1-propanol (1.0%), and 68 trace compounds (<1%)

**Table 2:** Genetic basis of canine olfaction and its implications for HRD dog performance.

Aspect	Findings in Dogs	Relevance for HRD	Ref.
OR gene repertoire	~1094 functional OR genes, one of the largest among mammals	Explains high olfactory acuity compared to humans (~400 ORs)	[21]
Genomic organization	~40 OR clusters, major loci on chromosomes 18 and 21	Suggests evolutionary expansion and specialization of olfaction	[22]
Genetic diversity	High SNP frequency, many leading to amino acid substitutions	Provides a molecular basis for inter-individual variability	[22]
Breed-related selection	Allelic variants associated with detection performance; enriched in scent-oriented breeds	May explain why retrievers/spaniels excel in HRD work	[23]
Expression variability	Transcript levels vary > 10,000-fold among ORs; age and environment modulate expression	Suggests dynamic adaptation of olfaction over the lifetime and the environment	[20]
Specialized receptors (TAARs)	Detect volatile amines (putrescine, cadaverine, trimethylamine) linked to decay	Supports the role of amines in the cadaveric odor signature	[24,25]

**Table 3:** The use of Sigma pseudo-scents simulating the odor of decomposing human remains for training working dogs across countries.

Country	Organization/Unit	Sigma Scent Type (SKU/Name)	Usage Context/Notes	Ref.
USA	K-9 Specialty Search School (North Franklin, Connecticut)—“K9 Connecticut”	Corpse Scent: Formulation I (P4304), Formulation II (P3929)	Training materials titled “Basic Cadaver Training Using Sigma Pseudo™…”. Direct, practical use for HRD training.	[99]
USA	FEMA US&R Canine Program	(HRD pseudo-odors permitted; no brand specified)	FEMA standards describe the use of odor training aids for LF/HRD; no public confirmation that Sigma is used.	[98]
UK	SARDA CanTech (Search And Rescue Dog Association—Canine Technical)	Drowned Victim (P7184)	Declared training of dogs using Sigma’s “Drowned Victim” scent; water-recovery HRD applications.	[101]
Germany	Vermisstefinden e.V.	Formulation I (P4304), Formulation II (P3929)	The organization states the active use of Merck (Darmstadt, Germany)/Sigma Pseudo Scents for training “Leichenspürhunde” (cadaver dogs).	[102]
Poland	Trainer market/distribution (e.g., Thor Working Dogs)	PSI (P4304), PSII (P3929), PSDV (P7184)	Availability and use in the HRD training community; police training programs exist, but do not specify brand.	[103]
Canada	Fisher Scientific Canada (distribution)	PSII (P3929), PSDV (P7184)	Official Merck/MilliporeSigma distribution; indirect evidence (availability to institutions), no public brand confirmation by specific services.	[104]

## Data Availability

No new data were created or analyzed in this study. Data sharing does not apply to this article.

## Supplementary Materials

The following supporting information can be downloaded at: https://www.mdpi.com/article/10.3390/molecules30204066/s1, Table S1: Volatile organic compounds (VOCs) reported from human remains across published studies.

## Abbreviations

The following abbreviations are used in this manuscript:

1D	One-dimensional
2D	Two-dimensional
ACEM	Automated Chemical Environment Monitor
ARF	Anthropology Research Facility
ATP	Adenosine triphosphate
cAMP	Cyclic adenosine monophosphate
CDD	Cadaver-detection dogs
CDI	Cadaver Decomposition Island
COVID-19	Coronavirus disease 2019
DHS	Department of Homeland Security
DMDS	dimethyl disulfide
DMS	dimethyl sulfide
DMTS	dimethyl trisulfide
DTI	diffusion tensor imaging
FBI	Federal Bureau of Investigation
FEMA	Federal Emergency Management Agency
FET-GC-ITD	Full Evaporation Technique-Gas Chromatography-Ion-Trap Detection
fMRI	functional magnetic resonance imaging
GABA	4-Aminobutanoic acid
GC	Gas Chromatography
GC × GC	Two-dimensional gas chromatography
GC-MS	Gas Chromatography-Mass Spectrometry
GPCR	G-protein-coupled receptor
HRD	human remains detection
HRV	heart-rate variability
ISO	International Organization for Standardization
LFD	life finding dogs
MDMPP	2-methyl-1-(1,1-dimethylethyl)-2-methyl-1,3-propanediyl propanoic acid
NOAA	National Oceanic and Atmospheric Administration
OB	olfactory bulb
OR	olfactory receptor
ORN	olfactory receptor neuron
P/T	Purge and Trap
PMI	postmortem interval
PSI	Sigma Pseudo™ Corpse Scent Formulation I
PSII	Sigma Pseudo™ Corpse Scent Formulation II
QBA	quantitative behavior assessment
SAR	Search and rescue
Sigma	Sigma Pseudo™ Corpse Scent
SPME	Solid-Phase Microextraction
TAAR	Trace amine-associated receptor
TOFMS	Time-of-flight mass spectrometry
VNO	vomeronasal organ
VOC	volatile organic compound
